# Supplementary figures and images for: Lipases secreted by a gut bacterium inhibit arbovirus transmission in mosquitoes
Source: PLoS Pathog. 2022 Jun 9;18(6):e1010552. doi: 10.1371/journal.ppat.1010552 (PMC9182268; doi:10.1371/journal.ppat.1010552)

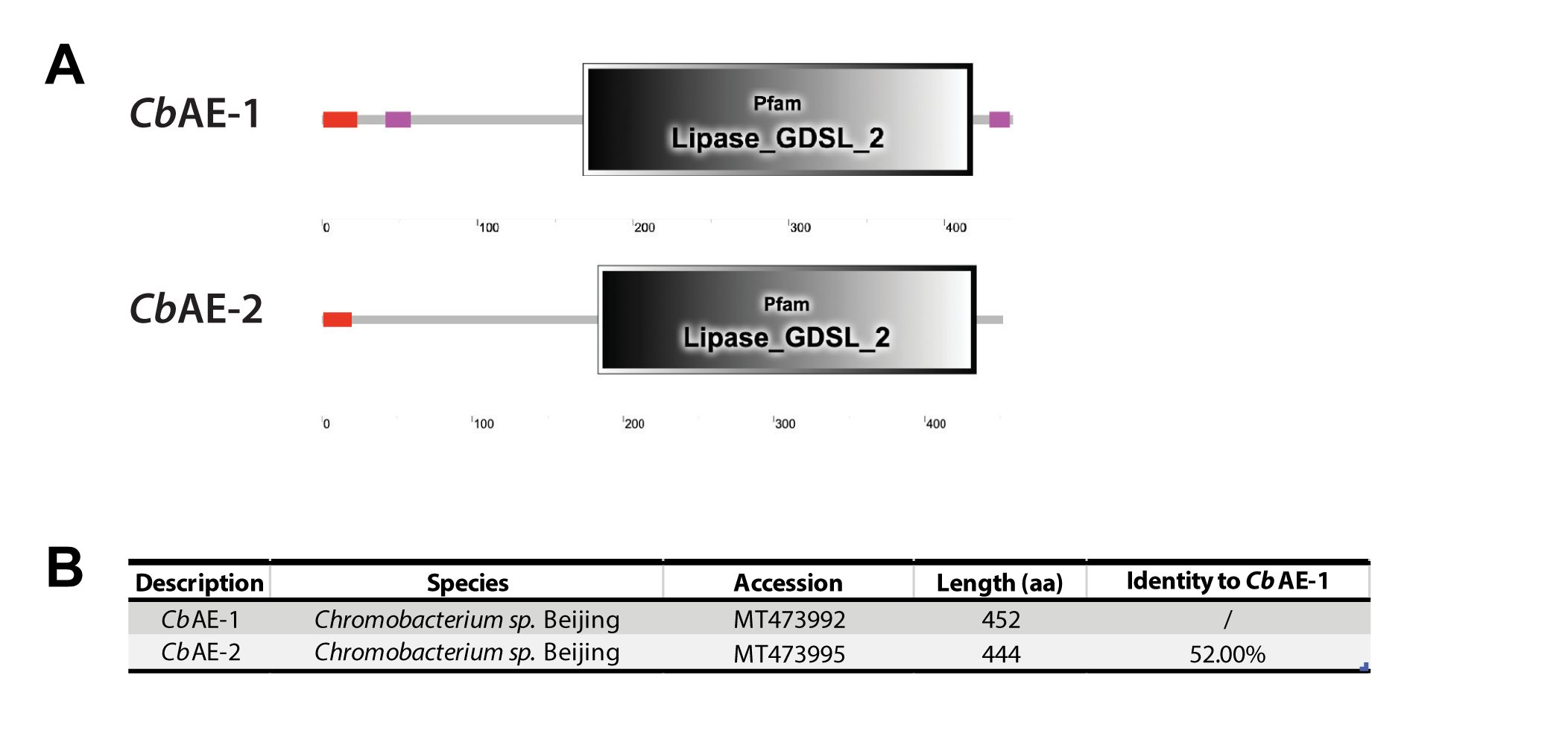

Supplement: S1 Fig — (A) Conserved domains of CbAE-1 and CbAE-2 protein sequences were analyzed using a Simple Modular Architecture Research Tool (SMART). (B) Sequence comparison of CbAE-1 and CbAE-2 was performed using the Basic Local Alignment Search Tool (BLAST) on the NCBI website with the program “Needleman-Wunsch alignment of two sequences”. (TIF) [file ppat.1010552.s001.tif]

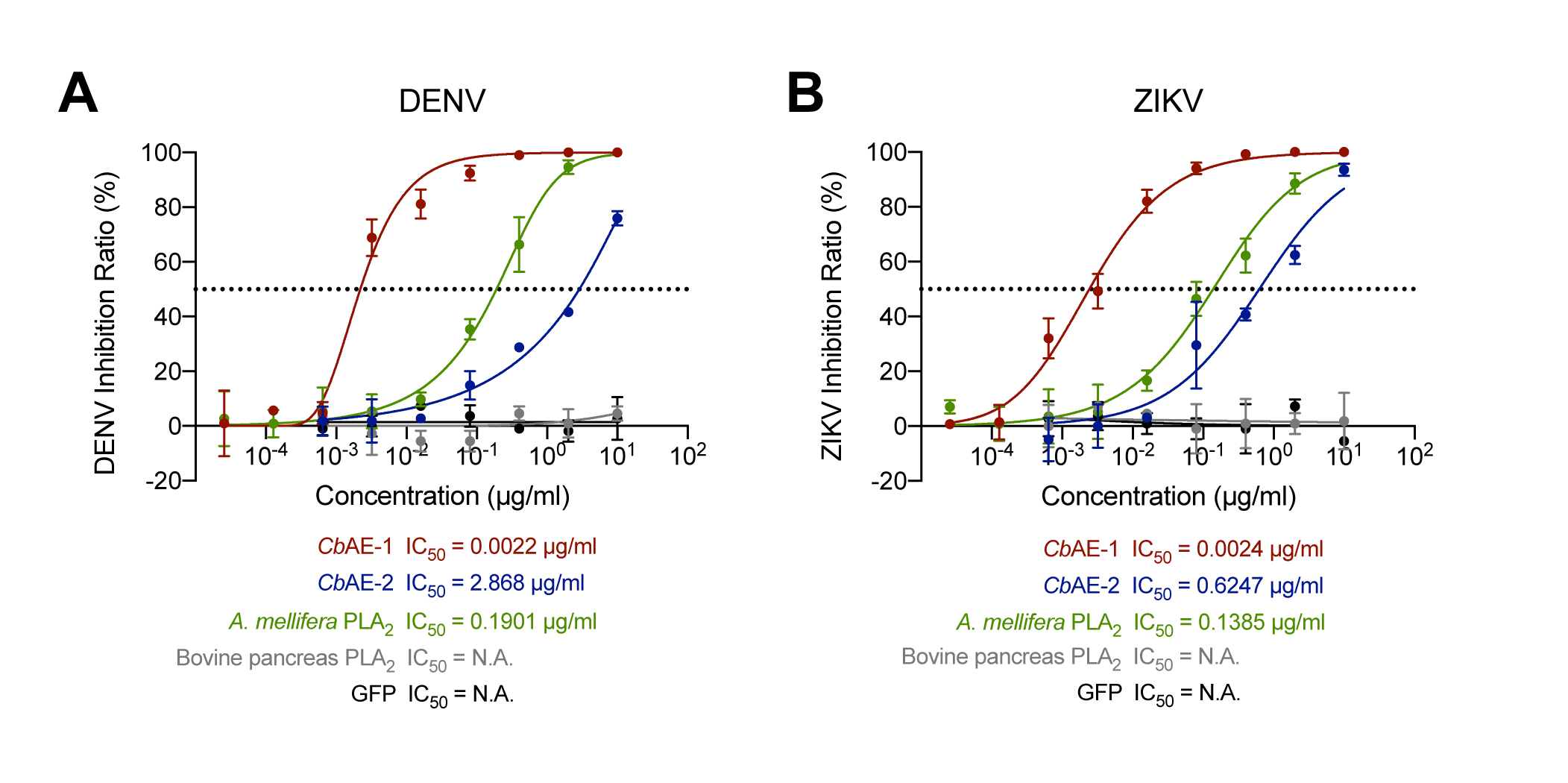

Supplement: S2 Fig — (A, B) Inhibition curves of CbAE-1, CbAE-2, A.mellifera PLA2 and bovine pancreas PLA2 against DENV (A) and ZIKV (B). Standard plaque reduction neutralization tests (PRNTs) were performed. Serial concentrations of proteins were mixed with 50 PFU of DENV or ZIKV in VP-SFM medium to perform standard plaque reduction neutralization tests (PRNTs). GFP was used as negative controls. (TIF) [file ppat.1010552.s002.tif]

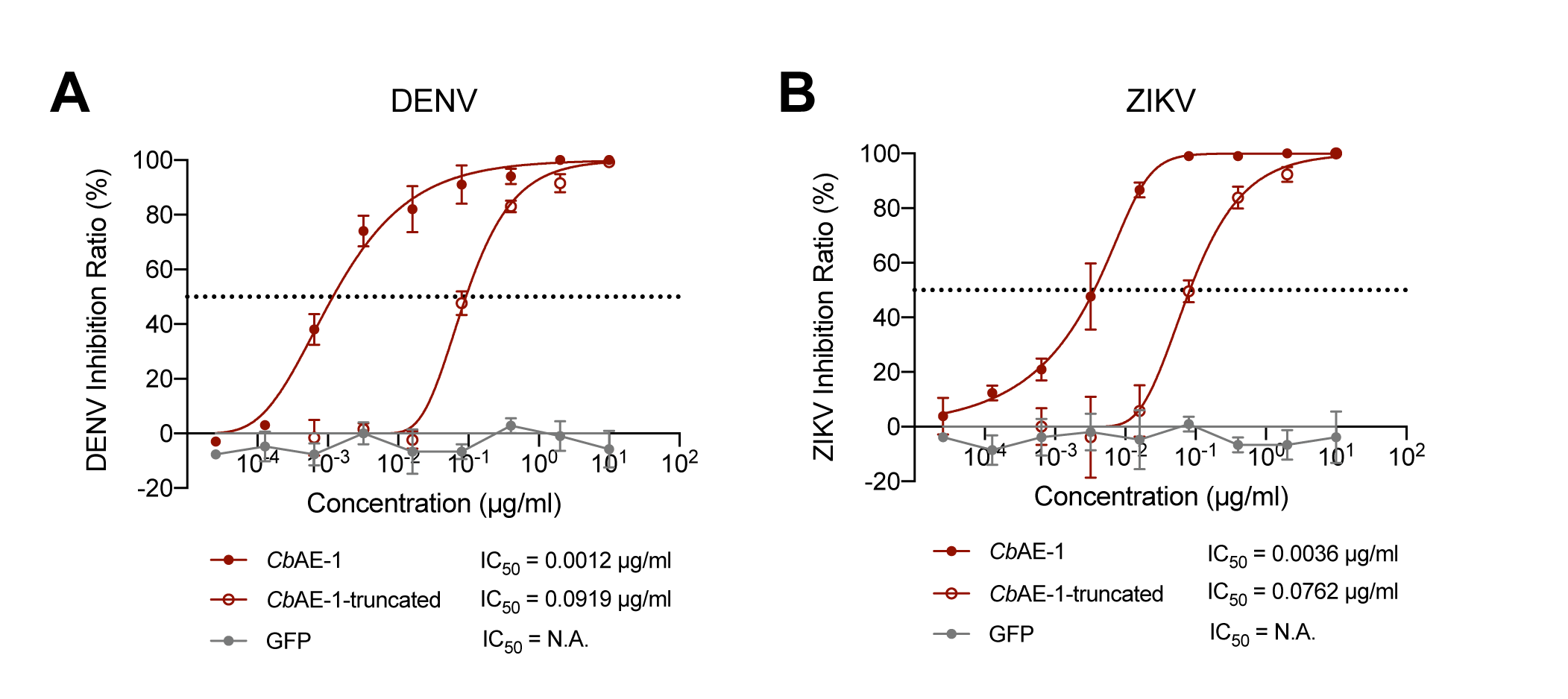

Supplement: S3 Fig — (A, B) Inhibition curves of CbAE-1 and CbAE-1-truncated against DENV (A) and ZIKV (B). Standard plaque reduction neutralization tests (PRNTs) were performed. Serial concentrations of CbAE-1 and CbAE-1-truncated were mixed with 50 PFU of DENV or ZIKV in VP-SFM medium to perform standard plaque reduction neutralization tests (PRNTs). GFP was used as negative controls. (TIF) [file ppat.1010552.s003.tif]

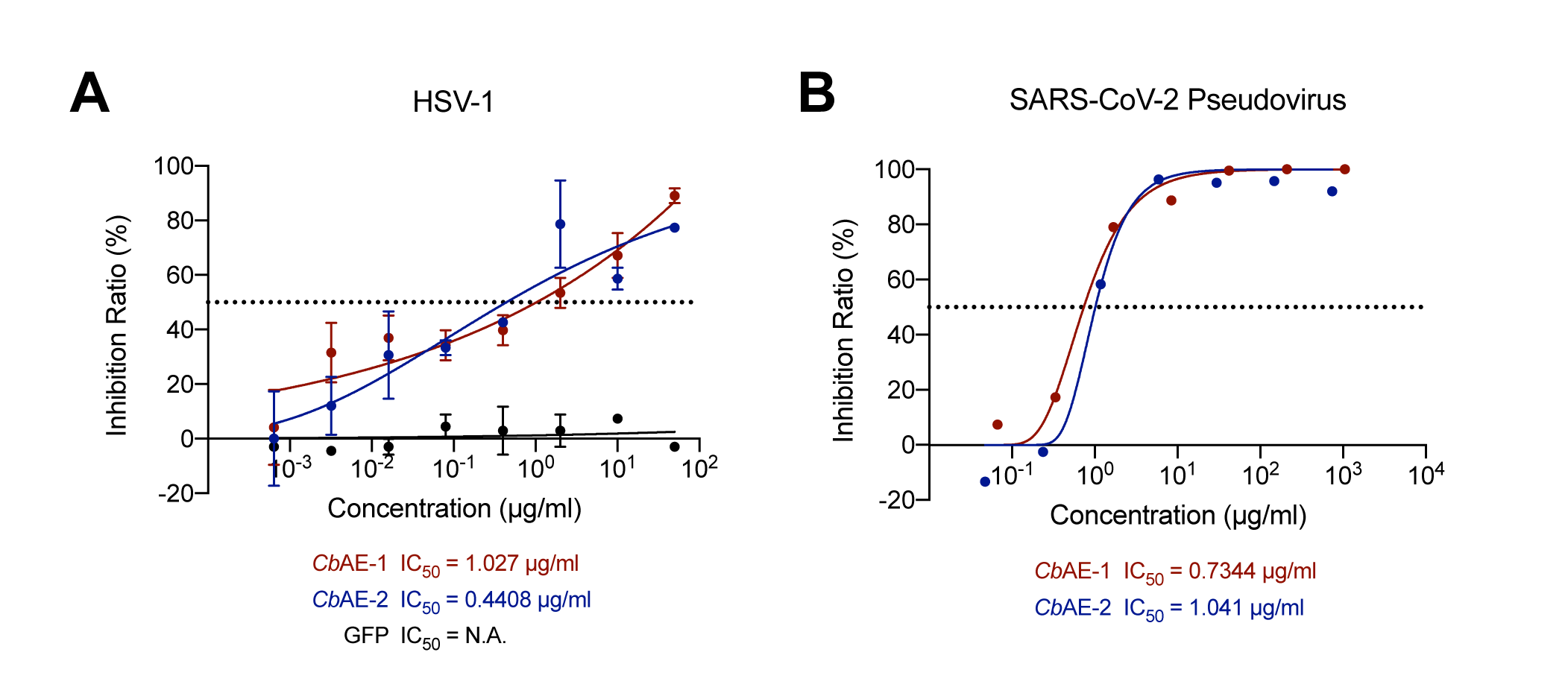

Supplement: S4 Fig — (A, B) Inhibition curves of CbAE-1 and CbAE-2 against HSV-1 (A) and SARS-CoV-2 pseudovirus (B). Standard plaque reduction neutralization tests (PRNTs) (A) or luciferase-based neutralization assays (B) was performed. GFP was used as a negative control. (TIF) [file ppat.1010552.s004.tif]

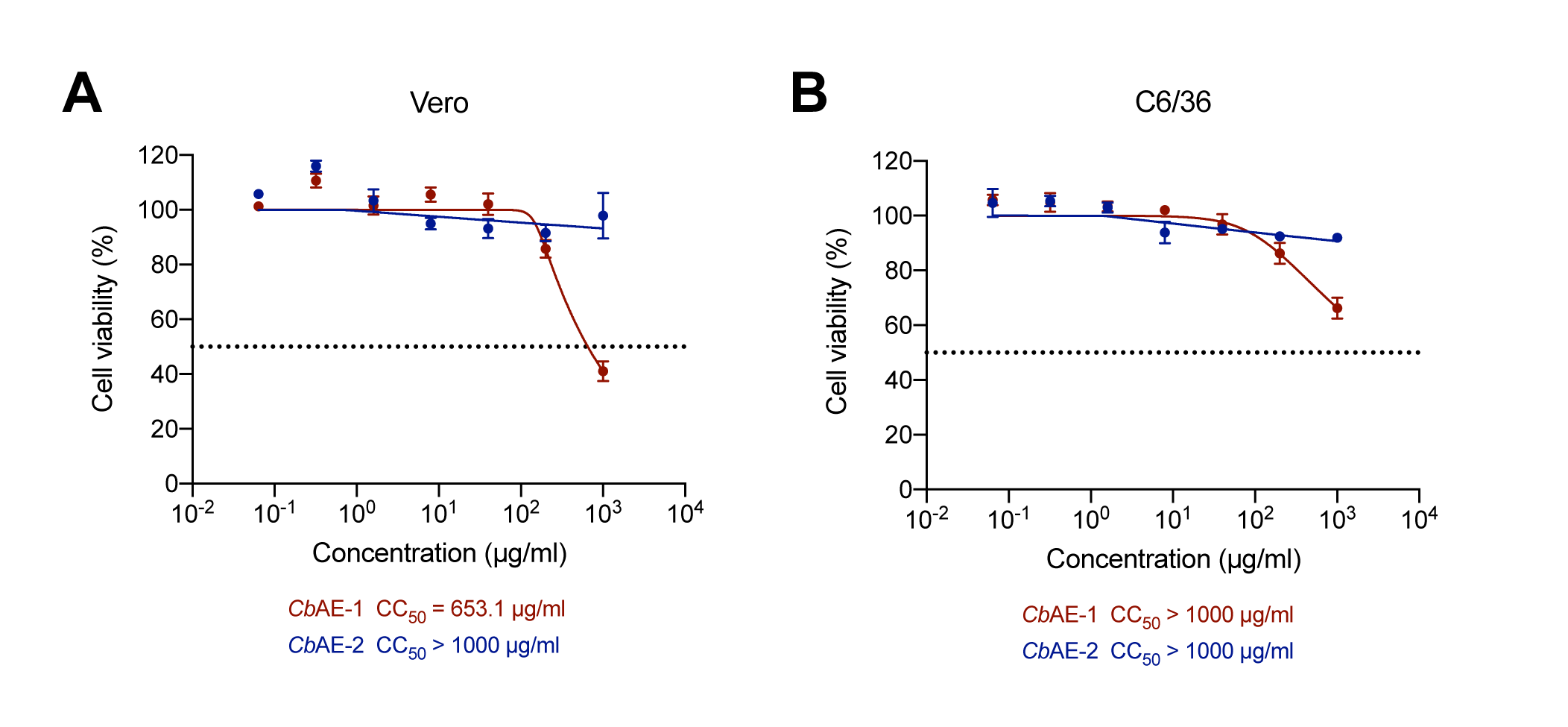

Supplement: S5 Fig — (A, B) Cytotoxicity of CbAEs to Vero cells (A) and C6/36 cells (B) was measured by MTT assays. (TIF) [file ppat.1010552.s005.tif]

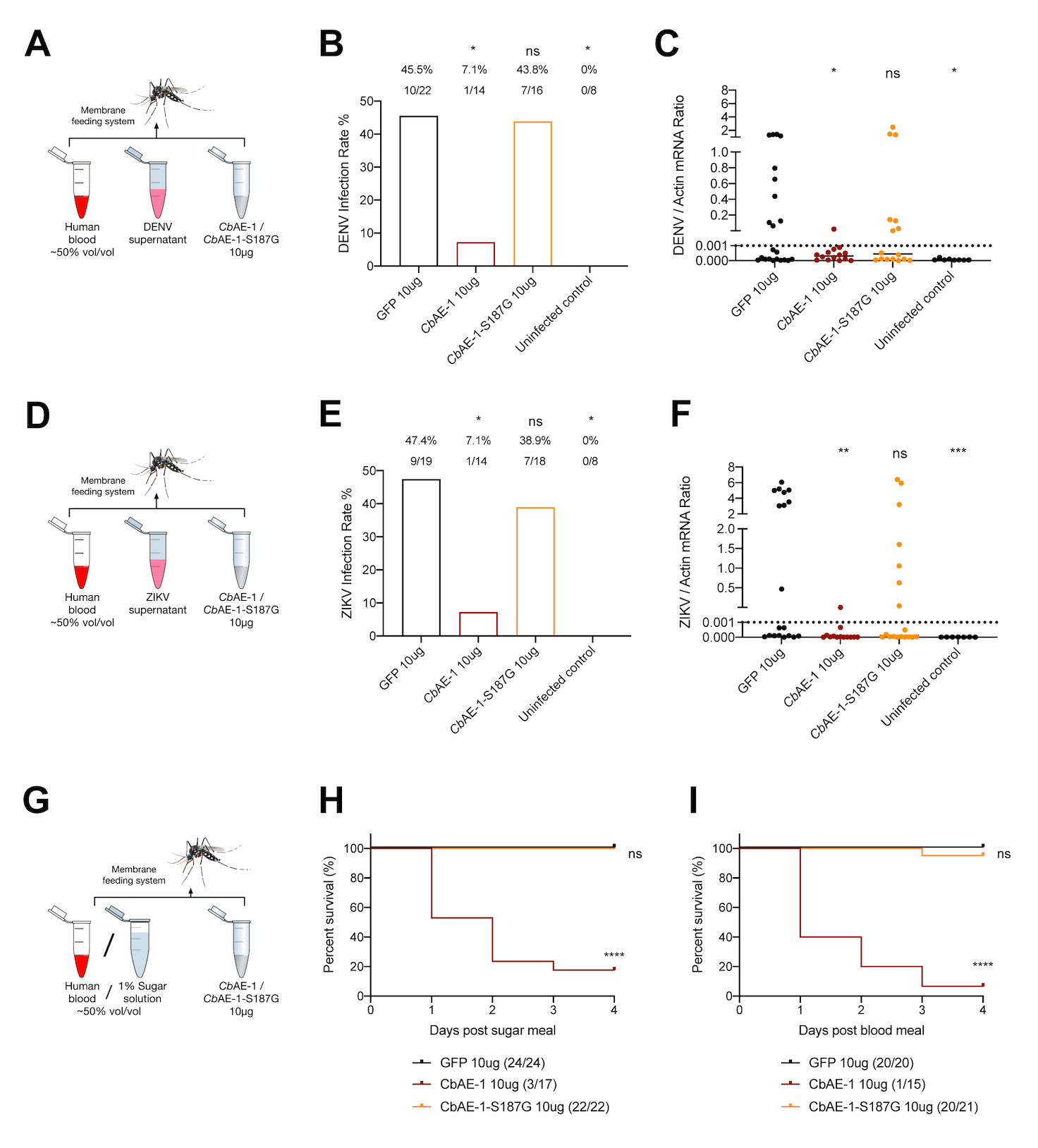

Supplement: S6 Fig — (A-F) The virucidal activity of CbAE-1 against DENV (A-C) and ZIKV (D-F) infection in A. aegypti is dependent on its lipase activity. (A, D) Schematic representation of the study design. A mixture containing human blood (50% v/v), 10 μg CbAE-1 or CbAE-1-S187G, and supernatant from DENV- (A) or ZIKV- (D) infected Vero cells was used to feed untreated A. aegypti via a membrane blood feeding system. Mosquito infectivity was determined by RT–qPCR at 8 days post blood meal. The final DENV or ZIKV titre was 1 × 10^5^ PFU/mL for oral infection. (B, E) Mosquito infection rate after oral supplementation of CbAE-1 or CbAE-1-S187G with DENV (B) or ZIKV (E). (C, F) Mosquito viral load after oral supplementation of CbAE-1 or CbAE-1-S187G with DENV (C) or ZIKV (F). (G-I) The entomopathogenic activity of CbAE-1 in A. aegypti is dependent on its lipase activity. (G) Schematic representation of the study design. A mixture containing 1% sugar solution (H) or human blood (I) (50% v/v) and 10 μg CbAE-1 or CbAE-1-S187G (50% v/v) was used to feed untreated A. aegypti via a membrane blood feeding system. Mosquito mortality was observed over 4 days. (B, C, E, F, H, I) The number of infected mosquitoes relative to total mosquitoes is shown at the top of each column (B, E). Differences in the infectivity ratio were compared using Fisher’s exact test (B, E). A nonparametric Mann–Whitney test was used for the statistical analysis (C, F). The survival rates of mosquitoes were plotted using a Kaplan–Meier curve and were statistically analyzed using the log-rank (Mantel-Cox) test (H, I). P values were adjusted using the Benjamini–Hochberg procedure (B, E, H, I) or Dunnett’s test (C, F) to account for multiple comparisons. The P value represents a comparison between the control group and the other groups. *P < 0.05, ***P < 0.001, ****P < 0.0001, ns, not significant. The limit of detection is illustrated by dotted lines (C, F). Experiments consisted of at least three biological replicates with simila [file ppat.1010552.s006.tif]
