# Supplementary material for: Lipases secreted by a gut bacterium inhibit arbovirus transmission in mosquitoes
Source: PLoS Pathog. 2022 Jun 9;18(6):e1010552. doi: 10.1371/journal.ppat.1010552 (PMC9182268; doi:10.1371/journal.ppat.1010552)
Supplement: S1 Table — (DOCX) [file ppat.1010552.s007.docx]

**Table S1. Primers for SYBR Green RT-qPCR and gene cloning.**

| **Primers for SYBR Green qRT-PCR** | **Forward Primer** | **Reverse Primer** |
| --- | --- | --- |
| DENV | CATTCCAAGTGAGAATCTCTTTGTCA | CAGATCTCTGATGAATAACCAACG |
| ZIKV | CCGCTGCCCAACACAAG | CCACTAACGTTCTTTTGCAGACAT |
| Aedes aegypti Actin | GAACACCCAGTCCTGCTGACA | TGCGTCATCTTCTCACGGTTAG |
| **Primers for cloning into pET28a** | **Forward Primer** | **Reverse Primer** |
| gene4149 | CTAGGCTTAAATGCAGACCCTTTCT | TGCCTCGAGCGTCGTAGCTGG |
| gene1348 | CTAGGCTTAAATGTACTCATTCAGC | TGCCTCGAGCCTCCGCGGCC |
| gene0807 | CTAGGCTTAAATGAAAAAATTTGTATTTG | TGCCTCGAGCCTTGTACTGCAGA |
| gene0217 | CTAGGCTTAAATGAAAACCTTAGGC | TGCCTCGAGCGCGCAGTTGG |
| gene3946 | CTAGGCTTAAATGAAACCACAACTG | TGCCTCGAGCGGCCGGAGCC |
| gene4743 | CTAGGCTTAAATGTCCAAACCACTT | TGCCTCGAGCGAAGCTCAGCC |
| gene1720 | CTAGGCTTAAATGAAAATGAAGTTAGC | TGCCTCGAGCCCAAATGCGAATG |
| gene1128 | CTAGGCTTAAATGAAAGCTTTGATTC | TGCCTCGAGCGATCGAGTTCAG |
| gene2152 | CTAGGCTTAAATGAGGACGAAACAA | TGCCTCGAGCGTCGCCGCC |
| gene0229 | CTAGGCTTAAATGAAAAAAAGTCTGAT | TGCCTCGAGCGAACTTGTGTACG |
| gene2932 | CTAGGCTTAAATGAAAAAAGTCCTG | TGCCTCGAGCCTTCAGCTTGATC |
| gene2402 | CTAGGCTTAAATGAAAAAGAGTCTGA | TGCCTCGAGCGAATGCGTGGAT |
| gene3771 | CTAGGCTTAAATGAAATCGCTGCTA | TGCCTCGAGCGCGGCGGGC |
| gene1742 | CTAGGCTTAAATGACATTCACCGC | TGCCTCGAGCGCGGGACGGC |
| gene1590 | CTAGGCTTAAATGAAAATCCAGCTG | TGCCTCGAGCTTGCATCGGGC |
| gene3769 | CTAGGCTTAAATGAAACCTCAACTG | TGCCTCGAGCGCGGCGCGCC |
| **Primers for cloning *Cb*AE-1-S187G** | **Forward Primer** | **Reverse Primer** |
| *Cb*AE-1-S187G | CGGCCTGCTATTGCTGGGCCTTGCCCTG | TCGCCTCAGCGGCGGGCCAGGGC |
| **Primers for cloning *Cb*AE-1-truncated** | **Forward Primer** | **Reverse Primer** |
| *Cb*AE-1-truncated | CTAGGCTTAAGGCGACAGCCTGTCC | TGCCTCGAGCGCGGCGGGCCAGG |
