# Supplementary material for: Lipases secreted by a gut bacterium inhibit arbovirus transmission in mosquitoes
Source: PLoS Pathog. 2022 Jun 9;18(6):e1010552. doi: 10.1371/journal.ppat.1010552 (PMC9182268; doi:10.1371/journal.ppat.1010552)
Supplement: Attachment — Submitted filename: Response.docx [file ppat.1010552.s008.docx]

**Response to the Editor’s Comment:**

*Each of the reviewers had significant concerns about the manuscript. One of the reviewers mentioned that there were major deficiencies. A revised manuscript will be considered only after all the comments from the reviewers are addressed.*

**Answer:** We are grateful for the positive comments from the editor and reviewers. We have taken all of the comments into consideration and have revised our manuscript accordingly. We believe that our additional experiments, revised analyses, and modifications of the text have substantially improved the manuscript.

**Responses to Referee #1:**

**#1A.** *CbAE-1 and CbAE-2 have 52% identity, but CbAE-1 seems have much stronger antiviral activities. What makes this difference?*

**#1A Answer:** According to a previous study on three isoforms of phospholipase A2 (CB1, CB2 and PLA_2_-IC) from *Crotalus durissus terrificus* snake venom, while CB1 and CB2 share 91% and 97% identity with PLA_2_-IC, respectively, the enzymatic activities of these three isoforms differ significantly (Vieira et al., 2013, Biochimie, 95: 2365-2375). Another study of PLA_2_ from snake venom showed that B*l*K-PLA_2_ and B*l*D-PLA_2_ had 58% similarity in amino acid sequence but also differed in antiviral activity against dengue virus (Cecilio et al., 2013, Toxins, 5: 1780-1798). It is possible that certain regions in these lipases play an important role in regulating their specificity or enzymatic activity. The difference in specificity or enzymatic activity resulted in variation of antiviral activities. However, the exact mechanism underlying this difference still needs further investigation, and we aim to identify factors affecting their specificity and enzymatic activity in future studies. We have added the discussion above to the revised manuscript (Lines 238-247, Page 9).

**#1B.** *Since Chromobacterium bacterium and CbAE-1 and CbAE-2 are harmful to mosquitoes, can you estimate how much Chromobacterium bacterium are inside the mosquito gut in the real world.*

**#1B Answer:** We recognize the reviewer's concern and thank the reviewer for the suggestion. We estimated the relative abundance of *Chromobacterium* bacterium inside the midgut of mosquitoes by 16S rRNA gene sequencing. Briefly, *Aedes aegypti* were anesthetized in a 4 °C refrigerator and then surface-sterilized by dipping and shaking them in 75% ethanol for 2 min and rinsing them with 1× PBS twice for 1 min each. Midguts were then dissected from each individual mosquito over a sterile glass slide containing a drop of 1× PBS, transferred to a microcentrifuge tube containing 200 µl of sterile PBS and macerated for 30 sec. Three 10-fold serial dilutions were then plated on LB agar and kept at room temperature for 48 hr. The bacterial colonies were identified via 16S rRNA gene sequencing and comparisons (Djadid et al., 2011, PLoS One, 6: e28484; Ramirez et al., 2012, PLoS Negl. Trop. Dis., 6: e1561). The results showed that the absolute abundance of *Chromobacterium* bacterium inside the *Aedes aegypti* midgut was approximately 200 colonies per midgut, and the relative abundance was 0.22%. We have added the results (Lines 89-91, Page 4) and methods (Lines 438-445, Page 23) to the revised manuscript.

**#1C.** *The molecular weights of CbAE-1 and CbAE-2 is around 50kDa which means they are not easy to produce. Also, they will face the problems of salability and immunogenicity. Did you take any effort to make some truncated forms to get the minimum active form of these proteins? For example, did the lipase domain alone show any activity?*

**#1C Answer:** We recognize the reviewer's concern and thank the reviewer for the suggestion. We cloned and purified a truncated form of *Cb*AE-1 that only consists of its GDSL lipase domain (181AA-339AA). The primers used for generating *Cb*AE-1-truncated are listed in Table S1. The *Cb*AE-1-truncated is approximately 29 kDa. We then tested the antiviral activity of *Cb*AE-1-truncated against DENV and ZIKV. *Cb*AE-1 and GFP served as positive and negative controls, respectively. The lipase domain alone did show antiviral activity against both DENV and ZIKV. However, the half-inhibitory concentration (IC_50_) of *Cb*AE-1-truncated was 20-80 times higher than that of *Cb*AE-1, indicating impaired virucidal activity of *Cb*AE-1-truncated (Fig. S3). We have added these results to the revised manuscript (Lines 188-193, Pages 188-193).

**#1D.** *The data suggest that CbAEs have antiviral activities against DENV, ZIKV, JEV, YFV and SINV. The last one is not a flavivirus, could it have different mechanisms?*

**#1D Answer:** As suggested by the reviewer, we have tested the antiviral activity of *Cb*AE-1 and *Cb*AE-2 against SARS-CoV-2 pseudovirus and HSV-1. Both *Cb*AEs showed varying degrees of virucidal activity against them (Fig. S4). Since *Cb*AEs have shown antiviral activities against enveloped viruses or pseudovirus from the families Flaviviridae, Togaviridae, Coronaviridae, and Herpesviridae, it is rational to hypothesize that the virucidal activity of *Cb*AEs is mediated through the same mechanism of lipase activity. We have added these results to the revised manuscript (Lines 200-207, Page 8).

**#1E.** *Have you ever test whether CbAEs have antiviral activities against other enveloped viruses except for these mosquito-borne viruses? If so, this would be interesting because CbAEs have the potential as broad-spectrum antiviral drug candidates.*

**#1E Answer:** We thank the reviewer for the suggestion. We tested the antiviral activity of *Cb*AE-1 and *Cb*AE-2 against SARS-CoV-2 pseudovirus and HSV-1. Both *Cb*AEs showed varying degrees of virucidal activity against them, and the half-inhibitory concentrations (IC_50_) of *Cb*AEs against them are listed in the figures (Fig. S4). It would be interesting to further modify or engineer *Cb*AEs into better broad-spectrum antiviral drug candidates, and we are aiming to investigate this subject in future studies. We have added these results to the revised manuscript (Lines 200-207, Page 8).

**#1F.** *Considering CbAE-1/-2 disrupt the lipid associate membranes, have you ever tested the cytotoxic effect of these proteins to mammalian cells?*

**#1F Answer:** We recognize the reviewer's concern and thank the reviewer for the suggestion. We tested the cytotoxicity of *Cb*AE-1 and *Cb*AE-2 in Vero cells and C6/36 cells. Briefly, the cell viability was measured by the MTT [3-(4,5-dimethylthiazol-2-yl)-2,5-diphenyl tetrazolium bromide] (M8180, Solarbio) method. Confluent cell monolayers contained in 48-well plates were exposed to different concentrations of the *Cb*AEs for 24 hr at 37 °C. Then, a final concentration of 0.5 mg/ml MTT was added to each well. After 4 hr of incubation at 37 °C, the supernatant was removed, and 250 μl of dimethyl sulfoxide (DMSO) was added to each well to solubilize the formazan crystals. After shaking for 10 min, the absorbance was measured at 490 nm. The concentration of each protein necessary to reduce cell viability by 50% (CC_50_) was calculated by comparison with the untreated cells using a sigmoidal nonlinear regression function to fit the dose–response curve in GraphPad Prism 8.0 (GraphPad Software, USA). The half-cytotoxicity concentrations (CC_50_) of *Cb*AE-1 and *Cb*AE-2 in both cell lines are indicated in the figures (Fig. S5). Only *Cb*AE-1 showed toxicity at higher concentrations in Vero cells. At concentrations used in experiments generating IC_50_ curves, neither *Cb*AE-1 nor *Cb*AE-2 showed any cytotoxicity against Vero cells or C6/36 cells. We have added the results (Lines 207-212, Page 8) and methods (Lines 716-726, Pages 32-33) above to the revised manuscript.

**Responses to Referee #2:**

**#2A.** *Cell lysate – how was it processed? Lots of effectors may be membrane bound and may not get into the midgut.*

**#2A Answer:** We recognize the reviewer's concern. The cultured bacteria were pelleted by centrifugation and washed twice in 1× PBS. The bacterial pellet was then resuspended in cold 1× PBS, and cell lysates were generated by probe sonication. Lysates were centrifuged at 8000 × g and 4 °C for 20 minutes to remove insoluble cell debris. The supernatant was used for mosquito feeding experiments or plaque assays on Vero cells. We have added the methods to the revised manuscript (Lines 464-485, Pages 24-25). According to protocols for isolating membrane proteins of gram-negative bacteria, the membrane proteins are collected by ultracentrifugation of cell lysates (Whitfield, Booth and Naismith, 2006, Int J Biol Macromol, 39: 10-14). In our experiments, the cell lysates generated by sonication were only centrifuged at 8000 × g to remove cell debris and were not subjected to ultracentrifugation. Thus, in our preparation, the membrane-bound effectors should still remain in the supernatant and were ingested into the mosquito midguts.

**#2B.** *Fig 1B/E – how was “dengue infection rate %” calculated?*

**#2B Answer:** After membrane feeding, fully engorged mosquitoes were transferred into new containers and maintained under standard conditions for an additional 8 days. When collecting mosquitoes at Day 8, dead mosquitoes were picked out and were not counted. Mosquitoes that were alive were subsequently euthanized and counted as the total number of mosquitoes. Each mosquito was subjected to RNA extraction, reverse transcription and qPCR. When detecting DENV or ZIKV by qPCR, mosquito samples above the limit of detection were considered infected and were counted. DENV/ZIKV infection rate % = number of infected mosquitoes/number of total collected mosquitoes × 100%. We have added a more detailed description of the methods to the revised manuscript (Lines 520-530, Page 26).

**#2C.** *Fig 1C/F – viral titer in whole mosquitoes?*

**#2C Answer:** Yes. Whole mosquitoes were subjected to RNA extraction and reverse transcription, and viral titers were determined by qPCR. We have clarified this in the revised manuscript (Lines 526-527, Page 26).

**#2D.** *Figure 1G-I – “bacterial suspension” – does it include supernatant also, or bacteria were spun down and resuspended? In other words, were secreted metabolites included in this suspension?*

**#2D Answer:** The bacterial suspension includes the original LB medium supernatant used to culture the bacteria. The bacteria were not spun down and the secreted metabolites were included in the suspension. We have clarified that in the revised manuscript (Lines 464-485, Pages 24-25).

**#2E.** *Figure 1H and 1I – unclear from text or legend as to what the difference between these are?*

**#2E Answer:** In Figure 1H, the *Csp_BJ* bacterial suspension was fed to mosquitoes with a 1% sucrose meal, while in Figure 1I, the 1% sucrose solution was replaced by human blood. When ingested with sucrose solution or blood, the growth of bacteria in the mosquito midgut may differ (Singh et al., 2022, Sci Rep, 12: 1495.). Thus, we observed the survival rate of mosquitoes in both conditions.

**#2F.** *Fig2A/B – When the authors carried out the incubation expts with the supernatant, upper retentate and lower filtrate with the 50PFU of virus, was a time zero sample quantified. This would be important to demonstrate that the activity of an enzyme rather than some non-specific inhibition is occurring.*

**#2F Answer:** We recognize the reviewer's concern and thank the reviewer for the suggestion. However, it was not possible to quantify a time zero sample due to the plaque assay procedure. When carrying out a plaque assay, the mixture containing virus needs to be incubated on Vero cells for at least one hour for the virus to infect the cells, which means that any “time zero sample” will still be incubated for at least one hour.

To address whether some nonspecific inhibition is occurring, we subjected the supernatant to heat inactivation at different temperatures for one hour, and then the heat-treated supernatant was incubated with viruses and titered on Vero cells. Incubation at 50 °C partially inactivated the antiviral activity of the supernatant, and incubation at 70 °C or 99 °C resulted in total loss of activity (Figure R1). Taken together, these data strongly suggest that the antiviral factors in the *Csp_BJ* culture supernatant were proteins rather than some small metabolites or nonspecific inhibitors.

**#2G.** ***(a)*** *Fig3F and 3G – was a positive control protein such as a known PLA2 and negative control such as BSA or GFP used? These would be important for comparison if generic lipase activity is the key effector here. Else, is it the specific activity of CbAE-1/2 compared to other lipases and if so what is the hypothesis?* ***(b)*** *Also, can the authors include a cytotoxicity assay for these protein IC50 curves since the protein is not removed following incubation with the virus?*

**#2G Answer:** We recognize the reviewer's concern and thank the reviewer for the suggestion.

**(a)** We have included PLA_2_ from *A. mellifera* honeybee venom (P9279, Sigma–Aldrich) with known antiviral activity and PLA_2_ from bovine pancreas (P6534, Sigma–Aldrich) in these experiments. We performed plaque reduction neutralization tests with *Cb*AE-1, *Cb*AE-2, *A. mellifera* PLA_2_, bovine pancreas PLA_2_ and GFP against DENV or ZIKV (*A. mellifera* PLA_2_ served as positive controls; GFP expressed using the same vector and purified using the same procedure as *Cb*AEs served as negative controls). *A. mellifera* PLA_2_ showed similar antiviral activities against the viruses as previously reported (Chen et al., 2017, Sci Rep, 7: 15931), and the half-inhibitory concentration (IC_50_) of *Cb*AEs was consistent with our previous results (Fig. S2). The bovine pancreas PLA_2_ did not show any antiviral activity (Fig. S2). The PLA_2_ superfamily is currently classified into six types, in which more than one-third of the members belong to sPLA_2_ (secreted PLA_2_), which is further divided into 10 groups and 18 subgroups (Murakami et al., 2015, J Lipid Res, 56: 1248-1261). These sPLA_2_s show functional variations and are involved in a wide range of biological functions and disease occurrences through lipid metabolism and signaling (Murakami et al., 2015, J Lipid Res, 56: 1248-1261). The sPLA_2_s from bacteria are classified into group XIV, while the sPLA_2_s from honeybee venom and bovine pancreas belong to group III and subgroup IB, respectively. It is possible that sPLA_2_s from different groups or subgroups differ in their ability to degrade the viral envelope. However, the exact mechanism underlying this difference still needs further investigation, and we aim to identify factors affecting their specificity and enzymatic activity in future studies. We have added the results (Lines 157-163, Pages 6-7) and discussion (Lines 247-256, Pages 9-10) above to the revised manuscript.

**(b)** We tested the cytotoxicity of *Cb*AE-1 and *Cb*AE-2 in Vero cells and C6/36 cells. The half-cytotoxicity concentrations (CC_50_) of *Cb*AE-1 and *Cb*AE-2 on both cell lines are indicated in the figures. Briefly, the cell viability was measured by the MTT [3-(4,5-dimethylthiazol-2-yl)-2,5-diphenyl tetrazolium bromide] (M8180, Solarbio) method. Confluent cell monolayers contained in 48-well plates were exposed to different concentrations of the *Cb*AEs for 24 hr at 37 °C. Then, a final concentration of 0.5 mg/ml MTT was added to each well. After 4 hr of incubation at 37 °C, the supernatant was removed, and 250 μl of dimethyl sulfoxide (DMSO) was added to each well to solubilize the formazan crystals. After shaking for 10 min, the absorbance was measured at 490 nm. The concentration of each protein necessary to reduce cell viability by 50% (CC_50_) was calculated by comparison with the untreated cells using a sigmoidal nonlinear regression function to fit the dose–response curve in GraphPad Prism 8.0 (GraphPad Software, USA). Only *Cb*AE-1 showed toxicity at higher concentrations in Vero cells. At concentrations used in experiments generating IC_50_ curves, neither *Cb*AE-1 nor *Cb*AE-2 showed cytotoxicity against Vero cells or C6/36 cells. We have added the results (Lines 207-212, Page 8) and methods (Lines 716-726, Pages 32-33) above to the revised manuscript.

**#2H.** *Materials and methods have 'mice' mentioned, but these data are not discussed in the paper.*

**#2H Answer:** We apologize for the mistake and have removed the part mentioning mice from the Methods section.

**#2I.** *Fig 3E - Protein expression and purification – how did the authors validate the identity of the purified proteins CbAE-1/2?*

**#2I Answer:** We recognize the reviewer's concern. The purified *Cb*AE-1 and *Cb*AE-2 proteins were identified by liquid chromatography–mass spectrometry (LC–MS) at the Protein Chemistry Technology Core, Tsinghua University. We have clarified this in the revised manuscript (Lines 628-630, Page 30). A detailed experimental procedure is described in the “Mass spectrometry” part of the Methods section (Lines 586-605, Pages 28-29).

**#2J.** *Fig 3F/G – were control proteins (positive control for lipase activity) and a negative control (non-lipase such as BSA or GFP) used in these experiments? If not, they will need to be included.*

**#2J Answer:** We recognize the reviewer's concern and thank the reviewer for the suggestion. We have included positive and negative controls in the experiments (please refer to **Answer** **#2G-a**) (Lines 157-163, Pages 6-7).

**#2K.** *Fig4F – can the authors provide a larger magnification of a select number of control and treated virus particles? It is quite difficult to see the structural differences and a zoomed in image will help.*

**#2K Answer:** We recognize the reviewer's concern and thank the reviewer for the suggestion. The original figure contains images of viral particles at magnification ratios of 30,000× and 120,000×. We have included images at a higher magnification ratio (240,000×) in the updated manuscript (Fig. 4F).

**#2L.** *Line 90 – “resist” is the wrong word to use. Additional checks of word usage would be good.*

**#2L Answer:** We thank the reviewer for the correction. We have revised this sentence to “We next aimed to understand the mechanism underlying the antiviral activity of *Csp_BJ* in mosquitoes” in the manuscript (Lines 109-110, Page 5).

**Responses to Referee #3:**

**#3A.** *The manuscript is not formatted as per journal guidelines. The following sections are not indicated in the main text: Author Summary, Introduction, Results, Discussion.*

**#3A Answer:** We thank the reviewer for the suggestion. We have revised the manuscript per the journal guidelines.

**#3B.** *Missing experimental procedures that should be added to the material and methods: Methods used to identify the Chromobacter Beijin from the gut of the mosquito.*

**#3B Answer:** We have included the following methods in the revised manuscript (Lines 438-445, Page 23):

“**Isolation and characterization of mosquito midgut bacteria.**

Mosquitoes were anesthetized in a 4 °C refrigerator and then surfaced-sterilized by dipping and shaking them in 75% ethanol for 2 min and rinsing them with 1× PBS twice for 1 min each. Midguts were then dissected from each individual mosquito over a sterile glass slide containing a drop of 1× PBS, transferred to a microcentrifuge tube containing 200 µl of sterile PBS and macerated for 30 sec. Three 10-fold serial dilutions were then plated on LB agar and kept at room temperature for 48 hr. The bacterial colonies were identified via 16S rRNA gene sequencing and comparisons (Djadid et al., 2011, PLoS One, 6: e28484; Ramirez et al., 2012, PLoS Negl. Trop. Dis., 6: e1561).”

**#3C.** *Sequencing methods and sequencing comparison methods.*

**#3C Answer:** We have included the following methods in the revised manuscript (Lines 447-462, Pages 23-24):

“**Whole genome sequencing of *Csp_BJ*.**

The genomic DNA library of *Csp_BJ* was constructed by using a QIAseq FX DNA Library Kit (Qiagen) according to the manufacturer’s instructions and then by paired-end sequencing using an Illumina NextSeq 500 platform with a 300-cycle NextSeq 500 reagent kit v2 (Illumina). The metagenomic samples were sequenced by single-end sequencing by using a 150-cycle NextSeq 500 Reagent Kit v2 (Illumina). The complete genome sequence of the strain was determined by using a PacBio Sequel (Pacific BioSciences) sequencer with a Sequel SMRT Cell 1M v2 (four/tray) and a Sequel sequencing kit v2.1 (Pacific BioSciences) for long-read sequencing (insert size, ≈10 kb). High-quality genomic DNA was used to prepare a SMRTbell library by using a SMRTbell Template Prep Kit 2.0 (Pacific Biosciences). The draft genome contigs were assembled by using A5-Miseq software with Illumina short reads (Coil et al., 2015, Bioinformatics, 31: 587-589). The circular genome sequence was constructed by using Canu version 1.4 (Koren et al., 2017, Genome Res, 27: 722-736), Minimap version 0.2-r124 (Li., 2016, Bioinformatics, 32: 2103-2110), racon version 1.1.0 (Vaser et al., 2017, Genome Res, 27: 737-746), and Circlator version 1.5.3 (Hunt et al., 2015, Genome Biol, 16: 294) with long read data. Error correction of the circular sequence was performed by using Pilon version 1.18 with short reads (Walker et al., 2014, PLoS One, 9: e112963). Annotation was performed in DFAST version 1.0.8 (Tanizawa et al., 2018, Bioinformatics, 34: 1037-1039) and NCBI-BLASTP/BLASTX against deposited *Chromobacterium* complete genome sequences.”

**#3D.** *No experimental method corresponding to Figure 1. G to I is described in this section.*

**#3D Answer:** We have included the following methods in the revised manuscript (Lines 577-584, Page 28):

“**Monitoring mosquito mortality rate after sugar/blood meals.**

Aseptic mosquitoes, human blood and a serial dilution of *Csp_BJ* culture suspension were prepared as described above. A mixture containing 1% sugar solution or human blood (50% v/v) and different dilutions of *Csp_BJ* bacterial suspension (50% v/v) was used to feed antibiotic-treated *A. aegypti* via a membrane feeding system (6W1, Hemotek). Mixtures containing 1% sugar solution or human blood (50% v/v) and fresh LB broth (50% v/v) served as negative controls. Fully engorged female mosquitoes were transferred into new containers and maintained under standard conditions. Mosquito mortality was observed over 4 days.”

**#3E.** *Method used to produce the mutated lipase*

**#3E Answer:** We have included the following methods in the revised manuscript (Lines 631-634, Page 30):

“The mutated lipase *Cb*AE-1-S187G was generated using a Q5 Site-Directed Mutagenesis Kit (E0554S, NEB) according to the manufacturer’s protocol and purified using the same procedures as described for *Cb*AEs. The primers used for generating *Cb*AE-1-S187G are listed in Table S1.”

**#3F.** *Plaque assay and mosquito feeding experiments of Figure 2*

**#3F Answer:** We have included the following methods in the revised:

“**Preparation of *Csp_BJ* culture suspension, culture supernatant, cell lysate, upper retentate and lower liquid filtrate.**

The concentration of the *Csp_BJ* culture suspension at OD600 = 1.0 was first determined by a colony-forming assay. Briefly, *Csp_BJ* was grown overnight in liquid LB at 30 °C and diluted to OD600 = 1.0 using fresh LB. Then, 10-fold serial dilutions of *Csp_BJ* culture suspension at OD600 = 1.0 were plated on LB agar and kept at 30 °C for 48 hr. The concentration of the *Csp_BJ* culture suspension at OD600 = 1.0 was calculated to be approximately 10^8^ CFU/ml*.* For mosquito feeding experiments, *Csp_BJ* was grown overnight in liquid LB at 30 °C. The overnight culture was diluted using fresh LB to OD600 = 1.0, which equals a concentration of approximately 10^8^ CFU/ml, and was further diluted to 10^4^-10^7^ CFU/ml using fresh LB.

To prepare different components of the *Csp_BJ* culture, *Csp_BJ* was cultured for 24 hr at 30 °C in liquid LB. The *Csp_BJ* suspension was then separated into bacterial cells and culture supernatant by centrifugation at 4000 × g and 4 °C for 10 minutes (Avanti J-26XP, Beckman). The cell-free culture supernatant was then filtered through a 0.22 µm filter unit (SLGP033NS) before use in mosquito feeding experiments or plaque assays. The pelleted bacteria were washed twice in 1× PBS and resuspended in cold 1× PBS, and cell lysates were generated by probe sonication. Lysates were centrifuged at 8000 × g and 4 °C for 20 minutes (Avanti J-26XP, Beckman) to remove insoluble cell debris and were then filtered through a 0.22 µm filter unit before use. The filtered cell-free culture supernatant was further separated using a 3 kDa cutoff filter (ACK5003PA, Millipore) by centrifugation at 4 °C (5810R, A-4-62, Eppendorf). The upper retentate (proteins and large peptides) or the lower liquid filtrate (small molecule compounds and short peptides) were transferred to new tubes for further experiments.” (Lines 464-485, Pages 24-25)

“**Identifying virucidal components of the *Csp_BJ* culture suspension.**

For mosquito feeding experiments, aseptic mosquitoes were prepared and maintained as previously described. **(a)** Either the bacterial cell lysate or the culture supernatant (25% v/v) was mixed with human blood (50% v/v) and DENV or ZIKV virus supernatant (25% v/v) for membrane blood feeding of antibiotic-treated *A. aegypti*. Fresh LB broth or the original *Csp_BJ* culturing suspension (25% v/v) mixed with human blood (50% v/v) and virus supernatant (25% v/v) served as negative or positive controls, respectively*.* The original *Csp_BJ* culturing suspension (25% v/v) mixed with human blood (50% v/v) and fresh VP-SFM (25% v/v) served as an uninfected control for viral genome detection in mosquitoes. **(b)** Either the upper retentate or the lower liquid filtrate (25% v/v) was mixed with human blood (50% v/v) and the virus supernatant (25% v/v) for membrane blood feeding of antibiotic-treated *A. aegypti*. Fresh LB broth or the unseparated cell-free culture supernatant (25% v/v) mixed with human blood (50% v/v) and virus supernatant (25% v/v) served as negative or positive controls, respectively*.* The unseparated cell-free culture supernatant (25% v/v) mixed with human blood (50% v/v) and fresh VP-SFM (25% v/v) served as an uninfected control for viral genome detection in mosquitoes. Mosquito infectivity was determined by RT–qPCR at 8 days post-blood meal. The final DENV or ZIKV titer was 1 × 10^5^ PFU/mL for oral infection.

For plaque assays: **(a)** Either the bacterial lysates or the culture supernatant (50% v/v) mixed with 50 PFU of DENV or ZIKV in VP-SFM medium (50% v/v) was incubated for 1 hr before being used for the infection of Vero cell monolayers. Fresh LB broth (50% v/v) mixed with virus-containing medium (50% v/v) served as a negative control. Either the bacterial lysates or the culture supernatant (50% v/v) mixed with fresh VP-SFM medium (50% v/v) served as uninfected controls. **(b)** Either the retentate or the filtrate (50% v/v) was mixed with 50 PFU of DENV or ZIKV in VP-SFM medium (50% v/v) and incubated for 1 hr before inoculation into Vero cell monolayers. Fresh LB broth (50% v/v) mixed with virus in VP-SFM medium (50% v/v) served as a negative control. The culture supernatant (50% v/v) mixed with virus in VP-SFM medium (50% v/v) served as a positive control. Either the retentate or the filtrate (50% v/v) mixed with fresh VP-SFM medium (50% v/v) served as uninfected controls. Plaque assays were performed as previously described. No plaques were observed in uninfected control wells. Inhibition ratios were calculated as follows: Inhibition ratio% = 100% - number of plaques in sample well/number of plaques in negative control well × 100%.” (Lines 532-562, Pages 26-27)

**#3G.** *In the material and methods section, there is a mention of mice experiments (Lines 358-359), but no detailed experimental procedures are provided and there is no data linked to these experiments in the manuscript.*

**#3G Answer:** We apologize for the mistake and have removed the part mentioning mice from the Methods section.

**#3H.** *Negative and positive control are not indicated for experimental procedures:* ***(a)*** *Lanes 76-84;* ***(b)*** *85-87;* ***(c)*** *94-106;* ***(d)*** *159-167;* *429*

**#3H Answer:**

**(a)** For Lines 76-84, which refer to **Figure 1A-F**, we have added the following sentences to the revised manuscript (Lines 283-287, Pages 12-13):

“Mixtures containing human blood (50% v/v), fresh LB broth (25% v/v), and supernatant from DENV- or ZIKV-infected Vero cells (25% v/v) served as negative controls. A mixture containing human blood (50% v/v), *Csp_BJ* bacterial suspension (25% v/v), and fresh VP-SFM medium (25% v/v) served as an uninfected control.”

**(b)** For Lines 85-87, which refer to **Figure 1G-I**, we have added the following sentences to the revised manuscript (Lines 294-296, Page 13):

“Mixtures containing 1% sugar solution or human blood (50% v/v) and fresh LB broth (50% v/v) served as negative controls” (please refer to **Answer #3N-a**).

**(c)** For Lines 94-106, which refer to **Figure 2A-D**, negative and positive controls are clarified in the revised Methods section (please refer to **Answer #3F**) (Line 317 and 322, Page 14; Lines 532-562, Pages 26-27).

**(d)** For Lines 159-167 and Line 429, which refer to **Figure 4G, H** and **Figure 5**, we have added the following sentences to the revised manuscript (Lines 643-645, Page 30; Lines 682-684, Page 31):

“The same amount of GFP expressed using the same vector and purified using the same procedure as *Cb*AEs in replacement of *Cb*AE-1 or *Cb*AE-2 in the procedure served as negative controls.”

**#3I.** *Lines 60-61: this sentence is unclear. What do the authors mean by “secreted bacterial proteins…. that resist infections”?*

**#3I Answer:** We thank the reviewer for the correction. We have replaced “resist” with “inhibit” in the revised manuscript (Line 76, Page 4).

**#3J.** *Lines 70-76: Why did the authors not compare the sequence of the Csp_BJ with the Csp_P?*

**#3J Answer:** We thank the reviewer for the suggestion. We have added a comparison of *Csp*_*BJ* with *Csp*_*P* and revised the sentence to “Based on genomic comparison, *Csp_BJ* shares 99.48% identity with *Chromobacterium haemolyticum CH06-BL*, 99.42% identity with *Chromobacterium rhizoryzae* strain *JP2-74* or 99.52% identity with *Chromobacterium sp.* Panama” (Lines 93-95, Page 4).

**#3K.** *Lines 76 to 84:* ***(a)*** *A detailed experimental procedure is missing from the material and methods.* ***(b)*** *The infection rate of the positive controls was ~60% for DENV and ~50% for Zika. Why?* ***(c)*** *Also, what was the percent mortality in these assays?* ***(d)*** *Why there is a difference in the total number of mosquitoes between groups?*

**#3K Answer:** In Lines 76 to 84, serial dilutions of *Csp_BJ* culture suspension were mixed with DENV or ZIKV in blood meals for mosquito feeding assays, and the mosquito infection rates were determined by qPCR (Figure 1A-F).

**(a)** We have included the following methods to the revised manuscript (Lines 487-530, Pages 25-26):

“**Generation of antibiotic-treated mosquitoes.**

*Aedes aegypti* (Rockefeller strain) were maintained on a 10% sugar solution at 28 °C and 80% humidity with a 12-h light/dark cycle. Sterile cotton, filter paper, and sterilized nets were used to maintain the cages as sterilely as possible. For experiments utilizing aseptic mosquitoes, female mosquitoes were maintained on a 10% sucrose solution with 20 units of penicillin and 20 mg of streptomycin per mL (15070-063, Thermo Fisher Scientific) for 5 days. The effectiveness of the antimicrobial treatment was confirmed by colony-forming unit assays prior to blood feeding. Briefly, two mosquitoes from each cup were collected and decontaminated in 75% ethanol and rinsed in sterile PBS. Midguts were then dissected under aseptic conditions, transferred to a microcentrifuge tube containing 200 µl of sterile PBS and macerated for 30 sec. The mixture was then plated on LB agar and kept at room temperature for 48 h. Mosquitoes were confirmed as aseptic if no colonies were observed.

**Membrane blood feeding and detection of mosquito infection rate.**

The mosquitoes were starved for 24 hr to allow the antibiotics to be metabolized prior to membrane feeding. *Csp_BJ* was grown overnight in liquid LB at 30 °C. The overnight culture was diluted using fresh LB to OD600 = 1.0, which equals a concentration of approximately 10^8^ CFU/ml, and was further diluted to 10^4^-10^6^ CFU/ml. Fresh human blood from healthy donors was placed in heparin-coated tubes (367884, BD Vacutainer) and centrifuged at 1,000 × g and 4 °C for 10 min to separate plasma from blood cells. The plasma was heat-inactivated at 55 °C for 60 min. The separated blood cells were washed three times with PBS to remove the anticoagulant. The blood cells were then resuspended in heat-inactivated plasma. Mixtures containing human blood (50% v/v), different dilutions of *Csp_BJ* bacterial suspension (25% v/v) and supernatant from DENV- or ZIKV-infected Vero cells (25% v/v) were used to feed antibiotic-treated *A. aegypti* via a membrane feeding system (6W1, Hemotek). Mixtures containing human blood (50% v/v), fresh LB broth (25% v/v) and supernatant from DENV- or ZIKV-infected Vero cells (25% v/v) served as negative controls. Mixtures containing human blood (50% v/v), 10^6^ CFU/ml *Csp_BJ* bacterial suspension (25% v/v) and fresh VP-SFM medium (25% v/v) served as uninfected controls. We used approximately 1 ml of liquid mixture per feeder and allowed the mosquitoes to feed in a dark environment at 28 °C and 80% humidity for 30 min. A total of 20-40 mosquitoes were allowed to feed in each group (numbers may differ for certain experiments). Mosquitoes were then anesthetized by cooling them in a 4 °C refrigerator. Once the mosquitoes were fully anesthetized, they were transferred to a petri dish embedded in an ice bucket. Mosquitoes were sorted by looking carefully at the abdomens for any sign of a blood meal, and those that had not fed were euthanized and discarded. Fully engorged mosquitoes were put back into cardboard cups, provided a sugar meal (a piece of cotton soaked in 10% sucrose), and incubated under standard rearing conditions for 8 days. Every day, the mosquitoes were monitored for the mortality rate. At Day 8, dead mosquitoes were picked out and were not counted. Mosquitoes that were alive were subsequently euthanized and counted as the total number of mosquitoes. Each whole mosquito body was subjected to RNA extraction, reverse transcription and qPCR. When detecting DENV or ZIKV by qPCR, mosquito samples above the limit of detection were considered infected and were counted. DENV/ZIKV infection rate % = number of infected mosquitoes/number of total collected mosquitoes × 100%.”

**(b)** The infection rates of *Aedes aegypti* with DENV or ZIKV may vary due to different strains of mosquitoes or different titers of viruses used in the experiment. Infection rates of ~60% for DENV or ~50% for Zika by blood meals in positive controls have been commonly observed in our lab conditions (Zhu et al., 2019, Nat Microbiol, 4: 2405-2415; Liu et al., 2017, Nature, 545: 482-486) and in other studies (da Moura et al., 2015, Parasit Vectors, 8: 114; Perez-Restrepo et al., 2019, Archives of Virology, 164: 149-158; Zhu et al., 2021, Parasit Vectors, 14: 504).

**(c)** The percent mortality of mosquitoes in the experiments detecting virucidal activity of orally ingested *Csp_BJ* (Figure 1A, D) is shown in Figure R2 and was comparable with the results observed in Figure 1I.

**(d)** According to our procedure, only fully engorged mosquitoes and mosquitoes alive at Day 8 were counted as the total number of mosquitoes. The number of mosquitoes that ingested blood or sugar meals was usually different among groups. We have clarified this in the Methods section (Lines 520-523, Page 26). Thus, it is reasonable that there was a difference in the total number of mosquitoes between groups.

**#3L.** *Line 81: the word “prevalence” does not seem appropriate in this context.*

**#3L Answer:** We thank the reviewer for the correction. We have replaced “prevalence” with “infection rates” in the revised manuscript (Line 100, Page 4).

**#3M.** *Line 83: this sentence is unclear. The authors should explain how their results imply a “close relationship” between Csp_BJ and Csp_P strains.*

**#3M Answer:** We thank the reviewer for the correction and have removed this unclear statement from the manuscript.

**#3N.** *Lines 85-87:* ***(a)*** *What was used as negative control?* ***(b)*** *Why was 1% sugar solution used in this experiment?* ***(c)*** *How was the bacterial concentration range determined?* ***(d)*** *Why there is a difference in the total number of mosquitoes between groups?* ***(e)*** *Was the mosquito mortality observed in this assay comparable with the one observed in the previous feeding assay?* ***(f)*** *There is a ~ 10% mortality in the blood fed control and no mortality in the controls fed with sugar water. Why?*

**#3N Answer:** In Lines 85-87, mosquitoes were given *Csp_BJ* culture suspension mixed with either sugar meals or blood meals, and mosquito mortality rates were observed (Figure 1G-I).

**(a)** We have added the following sentences to the revised manuscript (Lines 294-296, Page 13): “Mixtures containing 1% sugar solution or human blood (50% v/v) and fresh LB broth (50% v/v) served as negative controls” (please refer to **Answer #3H-b**).

**(b)** In our previous studies, 1% sucrose solution was routinely used as a sugar meal for mosquito feeding assays (Zhu et al., 2017, Nat Commun, 8: 1262). A range of 1.5% to 2% sucrose solution for mosquito sugar meals was also used in other studies detecting mosquito mortality rates after feeding (Ramirez et al., 2014, PLoS Pathog, 10: e1004398; Day et al., 1994, J Med Entomol, 31: 611-617). Thus, we followed our commonly used protocol and mixed the *Csp_BJ* culture suspension with 1% sucrose solution in these experiments.

**(c)** We first determined the concentration of the *Csp_BJ* culture suspension at OD600 = 1.0 by a colony-forming assay. Briefly, 10-fold serial dilutions of *Csp_BJ* culture suspension at OD600 = 1.0 were plated on LB agar and kept at 30 °C for 48 h. The concentration of *Csp_BJ* culture suspension at OD600 = 1.0 was calculated to be approximately 10^8^ CFU/ml*.* For the experiments shown in Figure 1G-I, *Csp_BJ* was grown overnight in liquid LB at 30 °C. The overnight culture was diluted using fresh LB to OD600 = 1.0, which equals a concentration of approximately 10^8^ CFU/ml, and was further diluted to 10^4^-10^7^ CFU/ml using fresh LB. We have clarified how we determined the bacterial concentration range in the Methods section (Lines 464-473, Page 24).

**(d)** According to our procedure, only fully engorged mosquitoes and mosquitoes alive at Day 8 were counted as the total number of mosquitoes. The number of mosquitoes that ingested blood or sugar meals was usually different among groups. We have clarified this in the Methods section (Lines 520-523, Page 26). Thus, it is reasonable that there was a difference in the total number of mosquitoes between groups.

**(e)** The percent mortality of mosquitoes in the experiments detecting virucidal activity of orally ingested *Csp_BJ* (Figure 1A, D) is shown in Figure R2 and was comparable with the results observed in Figure 1I (please refer to **Answer #3K-c**).

**(f)** The difference of ~ 10% mortality in the blood-fed control and no mortality in the controls fed with sugar water may be due to the difference of the blood/sugar meal itself. Previous studies have already shown that the survival of *Aedes aegypti* differs when maintained on blood or sugar solution, and the survival rate of *Aedes aegypti* maintained on blood was lower than that on a sugar diet (Day et al., 1994, J Med Entomol, 31: 611-617; Canyon et al., 1999, J Med Entomol, 36: 301-308). This observation might explain the mortality difference in the blood-fed or sucrose solution-fed controls.

**#3O.** *Line 90: what do the authors mean by “Csp_BJ resists viral infection in mosquitoes”? The sentence is unclear. The word “resists” does not seem appropriate.*

**#3O Answer:** We thank the reviewer for the correction. We have changed this sentence to “We next aimed to understand the mechanism underlying the antiviral activity of *Csp_BJ* in mosquitoes” in the revised manuscript (Lines 109-110, Page 5).

**#3P.** *Lines 92-94: this sentence is unclear, what does “differential fragmentation” refer to?*

**#3P Answer:** We thank the reviewer for the correction. We have changed this sentence to “We therefore identified the bacterial effector(s) that modulate DENV and ZIKV infection by separating the *Csp_BJ* culturing suspension into different fractions” in the revised manuscript (Lines 112-113, Page 5).

**#3Q.** *Lines 94-106:* ***(a)*** *In this set of experiments, there is a difference in the total number of mosquitoes between groups and between experiments (DENV vs. ZIKV)? Why?* ***(b)*** *Also, what was fed to the negative and positive controls group of mosquitoes?*

**#3Q Answer:** In Lines 94-106, the *Csp_BJ* culture suspension was separated into different fractions and subjected to antiviral activity tests in mosquito feeding assays and plaque assays on Vero cells (Figure 2).

**(a)** According to our procedure, only fully engorged mosquitoes and mosquitoes alive at Day 8 were counted as the total number of mosquitoes. The number of mosquitoes that ingested blood or sugar meals was usually different among groups. We have clarified this in the Methods section (Lines 520-523, Page 26). Thus, it is reasonable that there was a difference in the total number of mosquitoes between groups.

**(b)** The negative and positive controls are indicated in the revised Methods section (please refer to **Answer #3F**) (Lines 532-562, Pages 26-27).

**#3R.** *In the Figure 2 legend (Lines 254-255), negative (LB broth) and positive (cell culture supernatant +virus) controls are mentioned. I think that a better negative control would be using the bacterial supernatant, but without the virus. How was the percent inhibition ratio calculated?*

**#3R Answer:** We recognize the reviewer’s concern. The original negative control used in our experimental design, which was fresh LB broth (50% v/v) mixed with virus-containing medium (50% v/v), was used to calculate the inhibition ratio (inhibition ratio% = 100% - number of plaques in sample well/number of plaques in negative control well × 100%). Indeed, an uninfected control using the bacterial supernatant, but without the virus, is still needed. Thus, we repeated the experiments in Figure 2G-J and added uninfected controls to the procedure. The uninfected controls were defined as follows: in Figure 2G, H, either the bacterial lysates or the culture supernatant (50% v/v) mixed with fresh VP-SFM medium (50% v/v) served as the uninfected control; in Figure 2I-J, either the retentate or the filtrate (50% v/v) mixed with fresh VP-SFM medium (50% v/v) served as the uninfected control. As a result, no plaques should be observed in the uninfected control wells. We have clarified the controls used and the calculation method for the percent inhibition ratio in the revised manuscript (Lines 532-562, Pages 26-27). For a detailed description of the experimental procedures, positive and negative controls and the calculation method for the percent inhibition ratio, please refer to **Answer #3F**.

**#3S.** *Line 100: the word “prevalence” does not seem appropriate.*

**#3S Answer:** We thank the reviewer for the correction. We have replaced “prevalence” with “infection rates” in the revised manuscript (Line 120, Page 5).

**#3T.** *Lines 115-130:* ***(a)*** *What was the expected size of the expressed proteins?* ***(b)*** *What was used as negative/positive control in the assays of Figure 3-C-D-F-G?*

**#3T Answer: (a)** We have added the expected size of each expressed protein to the table in Figure 3A in the revised manuscript.

**(b)** For the PRNT tests of Figure 3C, D, F, G, serial concentrations of GFP expressed using the same vector and purified using the same procedure as *Cb*AEs (50% v/v) mixed with virus in VP-SFM medium (50% v/v) served as negative controls. A total of 1 μg of purified recombinant protein (Figure 3C, D) or 10 μg of *Cb*AE-1 or *Cb*AE-2 (Figure 3F, G) (50% v/v) mixed with fresh VP-SFM medium (50% v/v) served as the uninfected control. No plaques should be observed in the uninfected controls. Inhibition ratios were calculated as follows: inhibition ratio% = 100% - number of plaques in sample well/number of plaques in negative control well × 100%. We have clarified this in the revised manuscript (Lines 643-653, Page 30).

**#3U.** *Line 132: The sentence is unclear. The word “resists” does not seem appropriate.*

**#3U Answer:** We thank the reviewer for the correction. We have replaced “resist” with “inhibit” in the revised manuscript (Line 152, Page 6).

**#3V.** *Lines 138-139: How was the S187G mutation produced? There is no information in the material and methods.*

**#3V Answer:** We have included the following methods in the revised manuscript (Lines 631-634, Page 30):

“The mutated lipase *Cb*AE-1-S187G was generated using a Q5 Site-Directed Mutagenesis Kit (E0554S, NEB) according to the manufacturer’s protocol and purified using the same procedures as described for *Cb*AEs. The primers used for generating *Cb*AE-1-S187G are listed in Table S1.”

**#3W.** *Lines 144-145: Why was the BSA used as control? I think an unrelated protein expressed using the same vector and purified using the same procedure as the two lipases should be a more appropriate negative control.*

**#3W Answer:** We recognize the reviewer's concern and thank the reviewer for the suggestion. In Lines 144-145, a viral RNA exposure assay was described (Figure 4B, C). We agree with the reviewer’s suggestion that an unrelated protein expressed using the same vector and purified using the same procedure as the two lipases should be a more appropriate negative control. Thus, we have prepared GFP expressed using the same vector and purified using the same procedure as *Cb*AEs and repeated the experiments in Figure 4B and 4C using GFP as the negative control. We have clarified this in the Methods section (Lines 697-699, Page 32) of the revised manuscript.

**#3X.** *Lines 159-167: what was used as a control in these experiments?*

**#3X Answer:** We have added the following sentences to the revised manuscript (Lines 643-645, Page 30; Lines 682-684, Page 31):

“The same amount of GFP expressed using the same vector and purified using the same procedure as *Cb*AEs in the replacement of *Cb*AE-1 or *Cb*AE-2 in the procedure served as the negative control” (please refer to **Answer #3H-d**).

**#3Y.** *Line 162: the word “catalytic” does not seem appropriate.*

**#3Y Answer:** We thank the reviewer for the correction. We have replaced “catalytic” with “enzymatic” in the revised manuscript (Line 195, Page 8).

**#3Z.** *Lines 169-173:* ***(a)*** *There is a difference in the total number of mosquitoes between groups and between experiments (DENV vs. ZIKV)? Why?* ***(b)*** *Also, the infection rate is very low for the BSA control in DENV (33%), why?*

**#3Z Answer:** In Lines 169-173, the virucidal and entomopathogenic activities of *Cb*AE-1 and *Cb*AE-1-S187G were tested by mosquito feeding assays.

**(a)** According to our procedure, only fully engorged mosquitoes and mosquitoes alive at Day 8 were counted as the total number of mosquitoes. The number of mosquitoes that ingested blood or sugar meals was usually different among groups. We have clarified this in the Methods section (Lines 520-523, Page 26). Thus, it is reasonable that there was a difference in the total number of mosquitoes between groups.

**(b)** The infection rates of *Aedes aegypti* with DENV or ZIKV may vary due to slight variations in culturing conditions of different batches of mosquitoes or viruses. Infection rates ranging from ~30% to ~60% for DENV positive controls in mosquitoes have been observed in previous studies (Ramesh et al., 2019, iScience, 21: 564-576; Perez-Restrepo et al., 2019, Archives of Virology, 164: 149-158; Zhu et al., 2019, Nat Microbiol, 4: 2405-2415). We have repeated this experiment an additional two times, and the infection rates for the GFP control in DENV were 62.5% and 45.5%, respectively, which are comparable to the results in Figure 1. We have revised Figure 6B and C in the updated manuscript accordingly.

**#3AA.** ***(a)*** *Why did the authors used antibiotic-treated mosquitoes to test the lipases?* ***(b)*** *Would the lipases effect on virus infection rate in mosquitoes be the same in the presence of a normal gut microflora?*

**#3AA Answer:** We recognize the reviewer's concern and thank the reviewer for the suggestion.

**(a)** The virucidal and entomopathogenic activities of *Csp_BJ* were originally observed in antibiotic-treated mosquitoes. Thus, we continued to use antibiotic-treated mosquitoes to test the lipases.

**(b)** We have repeated the experiments in Figure 6 with untreated mosquitoes, and the results are shown in Fig. S6. The virucidal and entomopathogenic activities of *Cb*AE-1 and *Cb*AE-1-S187G were consistent with the results observed in aseptic mosquitoes. We have added these results to the revised manuscript (Lines 222-224, Page 9).

**#3AB.** *Lines 380-384: The blood feeding experiment needs more details. How much blood was used on the Hemoteck? How many mosquitoes were used/experiment? For how long were the mosquitoes left feeding, and under what conditions (temperature, humidity, light)? What method was used to transfer the fully fed mosquitoes into new containers? What was the mixture fed to the negative/positive controls?*

**#3AB Answer:** We recognize the reviewer's concern. For a more detailed experimental procedure of the blood-feeding experiments, please refer to **Answer #3K-a**. We have added the methods to the revised manuscript (Lines 500-530, Pages 25-26).

**#3AC.** *Line 388-389: the authors should describe the protocol used to test the mosquitoes to confirm the removal of gut bacteria. How were the mosquitoes processed? How many mosquitoes were used?*

**#3AC Answer:** We recognize the reviewer's concern. For a more detailed experimental procedure of removing mosquito midgut bacteria, please refer to **Answer #3K-a**. We have added the methods to the revised manuscript (Lines 487-498, Page 25).

**#3AD.** *Line 397:* ***(a)*** *Was the bacterium pelleted from the culture stock before the washes in PBS? If so, please indicate the centrifuge used (make and model) and centrifugation xg, time and temperature used.* ***(b)*** *Also, if the protein concentration step was done using a centrifuge, please indicate the centrifuge used (make and model) and centrifugation xg, time and temperature used.* ***(c)*** *How were the proteins purified from the SDS-PAGE gel before LC-MS?*

**#3AD Answer:** We have included the following methods in the revised manuscript:

**(a)** “*Chromobacterium sp.* Beijing was pelleted from the culture stock by centrifugation at 4000 × g and 4 °C for 30 minutes (Avanti J-26XP, Beckman). The pellet was then washed three times with cold PBS and suspended in cold VP-SFM medium” (Lines 587-589, Page 28).

**(b)** “The protein component of the bacterial supernatant was concentrated using an Ultra-15 centrifugal filter concentrator (UFC900396, Millipore) at 4000 rpm and 4 °C for 15 min repeatedly until the desired concentration was reached, and the proteins were then subjected to SDS–PAGE” (Lines 591-594, Page 28).

**(c)** “The SDS–PAGE gel was excised with a clean razor blade on a glass plate, and the pieces were placed into a 1.5 ml low-binding microcentrifuge tube with destaining buffer (50% acetonitrile, 50% 100 mM EPPS, pH 8.5 in ultrapure water). Gel pieces were dehydrated using acetonitrile, and in-gel enzymatic digestion was performed using trypsin at 37 °C overnight with shaking. Peptide extraction was performed using a solution of 1% formic acid and 75% acetonitrile in ultrapure water. Samples were vacuum centrifuged to dryness and reconstituted in a 5% formic acid and 5% acetonitrile buffer before analysis by liquid chromatography–mass spectrometry (LC–MS) at the Protein Chemistry Technology Core, Tsinghua University” (Lines 594-602, Pages 28-29).

**#3AE.** *Line 404: the gene amplification and cloning procedure needs more details: DNA extraction method, primers used, PCR cycle, cloning procedure.*

**#3AE Answer:** We have included the following methods in the revised manuscript (Lines 609-618, Page 29):

“Briefly, DNA extraction of *Csp_BJ* was performed using the E.Z.N.A. Bacterial DNA Kit (D3350, Omega Bio-Tek) according to the manufacturer’s protocol. The genes identified from mass spectrometry were then amplified with the primers described in Table S1. The amplification was performed using 2×TransTaq-T PCR SuperMix (AS122, Transgen) with a PCR cycle of 94 °C for 5 min, 35 cycles of 94 °C for 30 sec, 54 °C for 30 sec and 72 °C for 90 sec, and finally 72 °C for 10 min. DNA fragments were recovered and purified with an E.Z.N.A.® Gel Extraction Kit (D2500, Omega Bio-Tek) according to the manufacturer’s protocol. The recovered DNA fragments were then cloned into the pET-28a (+) expression vector using the pEASY-Basic Seamless Cloning and Assembly Kit (CU201, Transgen) according to the manufacturer’s protocol.”

**#3AF.** *Line 437: This paragraph needs more details: reaction mix used for dsRNA synthesis, RT-qPCR cycle.*

**#3AF Answer:** We have included the following methods in the revised manuscript (Lines 565-575, Pages 27-28):

“Total RNA was isolated either from homogenized mosquitoes or infected cell supernatant using a Multisource RNA Miniprep Kit (AP-MN-MS-RNA-250, Axygen) according to the manufacturer’s protocol. Total RNA was reverse transcribed to cDNA using an iScript cDNA Synthesis Kit (1708890, Bio–Rad) in a mixture of 1 μl of iScript Mix and 4 μl of RNA. Reverse transcription was performed at 25 °C for 5 min, 46 °C for 60 min and 95 °C for 1 min. Viral genomes were then quantified by qPCR using iTaq Universal SYBR Green Supermix (1725121, Bio–Rad) according to the manufacturer’s protocol using 1 μl of cDNA for each reaction. RT–qPCR was performed on a Bio–Rad CFX-96 Touch Real-Time Detection System. The RT–qPCR cycle was set to 95 °C for 2 min, 40 cycles of 95 °C for 10 sec and 60 °C for 30 sec, detection of melting curve and finally at 25 °C for 2 min. Primer sequences are shown in Table S1.”

**#3AG.** *Line 446: How was “CbAE-1-S187G” obtained?*

**#3AG Answer:** We have included the following methods in the revised manuscript (Lines 631-634, Page 30):

“The mutated lipase *Cb*AE-1-S187G was generated using a Q5 Site-Directed Mutagenesis Kit (E0554S, NEB) according to the manufacturer’s protocol and purified using the same procedures as described for *Cb*AEs. The primers used for generating *Cb*AE-1-S187G are listed in Table S1.”

**#3AH.** *Lines 454-455:* ***(a)*** *How was the RNase A neutralized?* ***(b)*** *What kits were used for RNA extraction and RT-qPCR.*

**#3AH Answer: (a)** According to the user manual of the Multisource RNA Miniprep Kit (AP-MN-MS-RNA-250, Axygen): “Tissues and cells are first lysed by Cell Lysis Buffer R-I, which also inactivates any indigenous RNase activity.” Thus, RNase A was neutralized in the first step of the RNA extraction.

**(b)** We have included the following methods in the revised manuscript (Lines 700-704, Page 32):

“Viral RNA was extracted using a Multisource RNA Miniprep Kit (AP-MN-MS-RNA-250, Axygen) according to the manufacturer’s protocol. RNase A was neutralized in the first step of the RNA extraction procedure by adding Buffer R-I to the mixture. RNA degradation was evaluated by RT–qPCR using the same kits and protocols as mentioned in the viral genome quantification section.”
